# Baseline SD-OCT characteristics of diabetic macular oedema patterns can predict morphological features and timing of recurrence in patients treated with dexamethasone intravitreal implants

**DOI:** 10.1007/s00592-020-01504-w

**Published:** 2020-02-29

**Authors:** Chiara M. Eandi, Daniele De Geronimo, Daniela Giannini, Maria Sole Polito, Gian Marco Tosi, Giovanni Neri, Yannick Le Mer, Monica Varano, Mariacristina Parravano

**Affiliations:** 1 Department of Surgical Sciences, University of Torino, C. Dogliotti 14, 10126 Turin, Italy; 2 Department of Ophthalmology, Jules-Gonin Eye Hospital, University of Lausanne, Fondation Asile des aveugles, Lausanne, Switzerland; 3 Department of Ophthalmology, Fondation Ophtalmologique A. De Rothschild, Paris, France; 4 IRCCS - Fondazione Bietti, Rome, Italy; 5 Ophthalmology Unit, Department of Medicine, Surgery and Neuroscience, University of Siena, Siena, Italy

**Keywords:** Diabetic macular oedema, Dexamethasone implant (DEX-I), Intravitreal treatment, Baseline characteristics, Recurrence, Spectral-domain optical coherence tomography

## Abstract

**Aims:**

To evaluate the timing and spectral-domain optical coherence tomography (SD-OCT) features of diabetic macular oedema (DME) recurrence according to baseline OCT patterns in patients treated with dexamethasone implant (DEX-I).

**Methods:**

This is a retrospective observational study (72 eyes/65 patients). Best-corrected visual acuity, timing of DME recurrence, and SD-OCT pattern [intraretinal cysts (IRC), IRC plus subretinal fluid (mixed), external limiting membrane (ELM), ellipsoid (IS/OS) layer integrity] were assessed at baseline and monthly until first DME recurrence.

**Results:**

Forty-two (58.3%) and 30 (41.6%) DME eyes had an IRC and mixed DME pattern at baseline, respectively. Twenty-four out of thirty mixed eyes (80%) relapsed without subretinal fluid. At baseline, mixed eyes showed similar changes in ELM and IS/OS (60 and 76.6% of eyes, respectively) versus IRC eyes (42.8 and 80.9% of eyes). After DME recurrence, more mixed eyes at baseline showed ELM and IS/OS changes (63.3 and 86.6%) than IRC eyes (50 and 76.2%). 33.3% of mixed eyes had DME recurrence at ≥ 6 months from first DEX-I implant versus 19% of IRC eyes.

**Conclusions:**

Mixed DME eyes were treated with DEX-I relapse later and more frequently without subretinal fluid than IRC eyes. SD-OCT characteristics of different DME patterns at baseline can predict morphological features and timing of DME recurrence.

## Introduction

Diabetic macular oedema (DME), a macular thickening secondary to diabetic retinopathy (DR), results from a blood–retinal barrier defect that leads to vascular leakage and fluid accumulation [1]. In patients with diabetes, DME is a leading cause of visual impairment and loss [2] and has been reported in almost 30% of patients with a duration of disease > 20 years [3].

DME has been related to the expression of several inflammatory factors, including vascular endothelial growth factor (VEGF), intercellular adhesion molecule-1 (ICAM-1), interleukin-6 (IL-6), monocyte chemotactic protein-1 (MCP-1), and leukostasis [4, 5]. Moreover, the expression of these factors has been related to both vascular permeability of the retina along with the severity of disease, thus confirming their important pathogenetic role [4]. While achieving control of glycemia is essential to limit the progression of DME, several treatment options for patients with DME are also in widespread use [2, 6]. Indeed, in the past decade, advances in the understanding of the pathogenesis of DME have led to the development of new therapies with anti-inflammatory action, especially steroids and VEGF inhibitors, which have resulted in several novel therapeutic applications [2, 7]. While intravitreal anti-VEGF agents have been shown to be effective in improving best-corrected visual acuity (BCVA) and decreasing central retinal thickness (CRT), it has been suggested that they should be used with caution due to possible systemic adverse events [2]. Moreover, they are not appropriate for all patients, and not all patients respond to anti-VEGF treatment; compliance to therapy also remains suboptimal due to the numerous injections required [8, 9].

In addition to anti-VEGF agents, in diabetic animal models, intravitreal corticosteroids have been shown to block the production of several inflammatory mediators, such as VEGF and ICAM-1, and inhibit leukostasis [10, 11]. In a clinical context, dexamethasone has been shown to have the highest relative efficacy among all corticosteroids that are routinely used to treat DME [6].

Dexamethasone intravitreal implant (DEX-I) is a matrix based on micronized dexamethasone embedded in a biodegradable copolymer of polylactic-co-glycolic acid that slowly releases the steroid into the vitreous over a period of months [12, 13]. DEX-I has been studied extensively in patients with DME. Based on the MEAD study, the Food and Drug Administration (FDA) and European Medicines Agency (EMA) approved DEX-I for the treatment of DME [14].

Several subsequent studies further demonstrated that DEX-I could improve BCVA and CRT in patients with DME and thus represents a viable treatment option [15, 16]. Similar results were obtained from the analysis of real-life data [17, 18]. A meta-analysis of four randomized clinical trials involving 521 eyes with DME reported that DEX-I is associated with improvements in BCVA that are non-inferior to anti-VEGF therapy, with superior anatomic outcomes at 6 months [6]. Moreover, compared to anti-VEGF agents, DEX-I requires fewer injections with no significant differences in the rates of adverse events, although there was some concern over raised intraocular pressure and cataract compared to anti-VEGF therapy. Given these favourable characteristics, DEX-I may be considered as first-choice therapy in selected cases, such as for pseudophakic eyes, failure of an anti-VEGF-agent, or in patients who are unwilling or unable to undergo frequent intravitreal injections [6].

To date, there is still limited evidence on the impact that individual characteristics of DME may have on the recurrence of DME following the implant of DEX-I. Some evidence has been presented that the DME morphologic subtypes, as defined by optical coherence tomography (OCT), may be associated with greater reductions in CRT in patients with DME. In particular, the serous retinal detachment (SRD) subtype has been associated with a greater reduction in the CRT than the diffuse retinal thickening (DRT) subtype [19], and in another study, the cystoid macular oedema (CME) and SRD subtypes showed greater reduction in CRT than the DRT subtype [20]. To shed further light on this aspect, we evaluated the spectral-domain (SD)-OCT morphological features of DME recurrence according to baseline OCT patterns in patients treated with DEX-I.

## Materials and methods

### Study design and patient population

This was a retrospective observational study. Informed consent was obtained from all subjects. All research procedures described in this study adhered to the tenets of the 1964 Declaration of Helsinki and its later amendments.

Clinical charts were retrieved from the four participating centres, and the pooled data were analysed. We included patients with a diagnosis of DME who had been treated with DEX-I during the period from 1 January 2017 to 30 June 2018 and followed at least until the first recurrence of DME at four referral centres (University of Torino, IRCCS-Fondazione Bietti in Rome, University of Siena, and Rothschild Foundation in Paris).

All patients had data relating to BCVA and SD-OCT features available at baseline and at each follow-up examination. BCVA was measured using the Early Treatment Diabetic Retinopathy Study (ETDRS) charts and reported as LogMar. Follow-up examinations included maximal answer (defined as macula dry or with the minimum amount of intra- or subretinal fluid) and information on DME recurrence (mean recurrence timing, characteristics of DME on SD-OCT). Baseline data also included the following: demographic data, diabetes duration, per cent glycated haemoglobin (HbA_1c_) level, and information on previous intravitreal treatment. Exclusion criteria were: macular oedema secondary to causes other than diabetes; previous treatment with intraocular corticosteroids; previous anti-VEGF intravitreal injections within the 6 months before treatment with the DEX implant; previous macular laser; and previous pars plana vitrectomy.

SD-OCT images were acquired with a Spectralis HRA + OCT instrument (Heidelberg Engineering, Heidelberg, Germany, version 6.4.7.0). The scanning protocol included a high-resolution 20° × 20° volume scan centred in the central macula. CRT was measured using the retina map pattern and the provided ETDRS grid in the central millimetre. Each section was obtained using ART (automatic real-time) eye tracking, and 16 scans were averaged to improve the signal-to-noise ratio. The following SD-OCT features were considered: CRT, DME pattern classified according to the presence of intraretinal cysts (IRC) or IRC plus subretinal fluid (mixed) pattern, the integrity of the external limiting membrane (ELM), and the ellipsoid junction (IS/OS).

The BCVA and SD-OCT characteristics were evaluated by two expert observers (CME and MP) at baseline and then monthly after DEX-I treatment until the first recurrence of DME. Patients were retreated according to a pro re nata (PRN) regimen if there was a recurrence of DME, defined as the presence of intra- or subretinal fluid on SD-OCT, also in the absence of visual impairment.

### Statistical analysis

The normal data distribution was tested using the one-sample Kolmogorov–Smirnov test. All continuous variables were expressed as mean ± standard deviation, while categorical variables as frequency and percentage. *T* test and Mann–Whitney test were performed as appropriate. Contingency tables (chi-square test) were used to investigate the relationship between pre- and post-recurrence DME pattern and between baseline DME pattern and pre- and post-recurrence morphological parameters (ELM and IS/OS). A one-way repeated measures analysis of variance (ANOVA) for each group was conducted to evaluate the null hypothesis that there is no change in functional and morphological parameter values when measured at baseline, maximal answer, and after recurrence in study groups. Post hoc tests were performed using the Bonferroni correction. Statistical evaluation was performed using SPSS (IBM SPSS Statistic 25). A *p* value < 0.05 was considered statistically significant.

## Results

### Baseline demographic characteristics

Considering pooled data from the four centres, baseline information and follow-up data were available on a total of 72 eyes from 65 patients with DME and receiving a DEX-I. The cohort included 39 males (60%) and 26 females (40%), with a mean age of 57.2 ± 8.2 years, mean duration of type 2 diabetes of 17.2 ± 8.8 years, and mean per cent HbA_1C_ at baseline of 8.2 ± 1.8 (66.0 ± 17.3 mmol/mol). Forty-three of the 72 eyes (59.7%) were phakic, and 29 (40.3%) were pseudophakic. Thirty-nine of the 72 eyes (54.2%) were naïve to treatment, and 33 of 72 eyes (45.8%) were switched from previous anti-VEGF treatment (Table 1).

**Table 1:** Demographic and baseline characteristics of patients

Characteristic	
Patients/eyes (*n*)	65/72
Male/female (*n*)	39/26
Age, mean ± SD (years)	57.2 ± 8.2
Duration of diabetes (years) mean ± SD	17.2 ± 8.8 (range 5–45)
HbA_1c_ % (mean ± SD)	8.2 ± 1.8^a^
Phakic/pseudophakic (*n* eyes)	43/29
Naïve/switched (*n* eyes)	39/33

*n* number, *SD* standard deviation, *HbA*_*1c*_ glycated haemoglobin

^a^66.0 ± 17.3 mmol/mol

### SD-OCT features

**Fig. 1:**
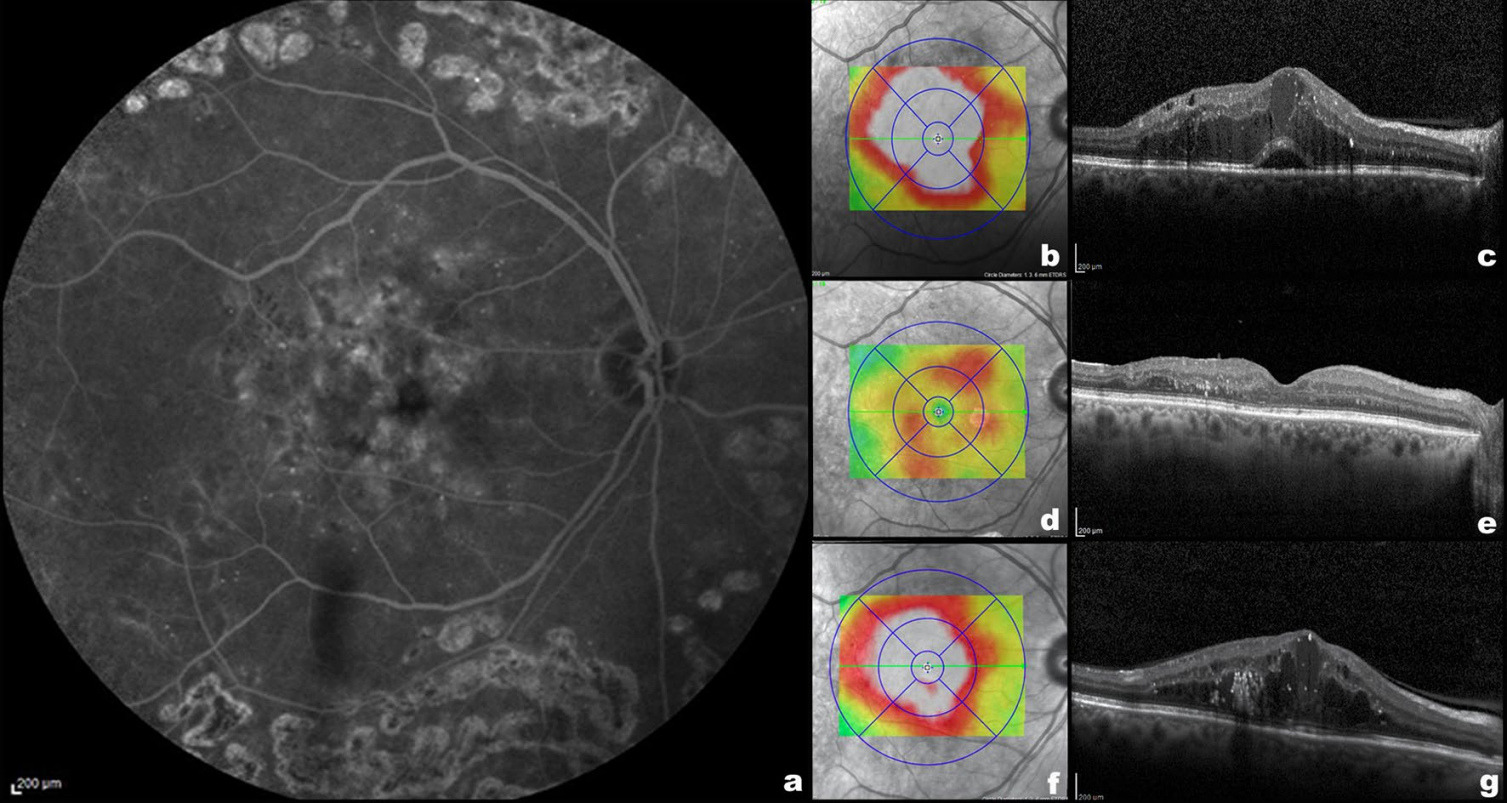
Left: Fluorescein angiography **a** of a patient with diabetic macular oedema before the treatment with dexamethasone intravitreal implants (DEX-I). Top right: Spectralis thickness map (**b**) and B-scan **c** of the same patient before the treatment with DEX-I, showing intraretinal cysts and subretinal fluid (mixed pattern). Middle right: Spectralis thickness map (**d**) and B-scan **e** of the same patient 2 months after the treatment with DEX-I showing a reduction in the macular thickness and no intra- or subretinal fluid. Bottom right: Spectralis thickness map (**f**) and B-scan **g** of the same patient 7 months after the treatment with DEX-I showing new diabetic macular oedema with intraretinal cysts without subretinal fluid

Forty-two (58.3%) and 30 (41.6%) eyes presented with an IRC and mixed DME pattern at baseline, respectively. Maximal answer (macula dry or with the minimum amount of intra- or subretinal fluid) timing was 1.83 ± 0.80 months for all patients, 1.83 ± 0.82 months for IRC group, and 1.83 ± 0.79 for the mixed group. Twenty-four of 30 mixed eyes (80%) treated with DEX-I relapsed without subretinal fluid (Table 2). A significant relationship was found between basal and post-recurrence DME pattern, X2 (1, *n* = 72) = 6.19, *p* = 0.013. A Mann–Whitney test showed that no differences were present in mean recurrence timing of baseline IRC and mixed groups (IRC group 5.74 ± 1.98 months; mixed group 6.27 ± 2.69 months, *p* = 0.168). However, a higher percentage of mixed eyes (33.3%) had a recurrence of DME after 6 months or longer after the first DEX-I implant in comparison with IRC eyes (19%) (Table 2). Figure 1 shows the SD-OCT features of a representative patient with mixed pattern DME before, 2 months after, and at the time of recurrence 7 months after treatment with DEX-I.

**Table 2:** Baseline DME pattern versus post-recurrence DME pattern and timing of recurrence

	Baseline DME pattern		
	IRC	Mixed	*p*
Post-recurrence DME pattern, eyes (%)			
IRC	41/42 (97.6)	24/30 (80.0)	0.013
Mixed	1/42 (2.4)	6/30 (20.0)	
Mean recurrence timing, months ± SD	5.74 ± 1.98	6.27 ± 2.69	0.625
> 6 months, eyes (%)	8/42 (19.0)	10/30 (33.3)	0.168
≤ 6 months, eyes (%)	34/42 (81.0)	20/30 (66.6)	

*DME* diabetic macular oedema, *IRC* intraretinal cysts, *SD* standard deviation

At baseline, mixed eyes showed similar changes in ELM and IS/OS (60 and 76.6% of eyes, respectively) compared with IRC eyes (42.8 and 80.9% of eyes, respectively). After a recurrence of DME, baseline mixed eyes showed greater changes in ELM and IS/OS (63.3% and 86.6% of eyes, respectively) compared with IRC eyes (50 and 76.2% of eyes, respectively) (Table 3).

**Table 3:** Spectral-domain optical coherence tomography (SD-OCT) characteristics by group

SD-OCT characteristics	IRC	Mixed	*p*
Baseline IS/OS changes, eyes (%)	34/42 (80.95)	23/30 (76.67)	0.659
Baseline ELM changes, eyes (%)	18/42 (42.86)	18/30 (60.00)	0.151
Post-recurrence IS/OS changes, eyes (%)	32/42 (76.20)	26/30 (86.70)	0.268
Post-recurrence ELM changes, eyes (%)	21/42 (50.00)	19/30 (63.30)	0.262
Mean baseline CRT (μm ± SD)	516 ± 136	600 ± 116	0.008
Mean post-recurrence CRT (μm ± SD)	462 ± 131	614 ± 150	0.006

*CRT* central retinal thickness, *ELM* external limiting membrane, *IRC* intraretinal cysts, *IS/OS* ellipsoid junction, *SD* standard deviation

By T test, a significant difference was found between basal CRT of the IRC and mixed groups at baseline (IRC 516 ± 136 μm; mixed 600 ± 116 μm; *p* = 0.008). Moreover, significant differences between groups were found in CRT at post-recurrence (IRC at baseline 462 ± 131 μm; mixed at baseline group 614 ± 150 μm; *p* = 0.006).

In both the IRC and mixed groups, ANOVA indicated a significant effect of time on CRT (*p* < 0.01). Post hoc tests indicated that pairwise differences in the IRC group were significant between baseline (516 ± 136 μm) and maximal answer (307 ± 76 µm) time (*p* = 0.009) and between maximal answer and post-recurrence (460 ± 135 μm) time (*p *< 0.01). In the mixed group, there was a significant decrease in CRT values between baseline (600 ± 116 µm) and maximal answer (336 ± 93 μm) time, as well as an increase between maximal answer and post-recurrence (499 ± 145 μm) time. The maximal answer for both groups was at month 2.

At baseline, treatment-naïve eyes showed fewer IS/OS changes than switched eyes in both subgroups of DME patterns (66.7% vs. 91.7% in IRC and 60% vs. 85% in the mixed group) (Table 4). After a recurrence of DME, a similar trend was observed: Naïve eyes showed fewer IS/OS changes than switched eyes in both subgroups of DME patterns (61.1% vs. 87.5% in IRC and 70% vs. 95% in the mixed group).

**Table 4:** SD-OCT changes of ELM and IS/OS layers by subgroup of patients (naïve, switched)

SD-OCT changes, eyes (%)	IRC			Mixed		
	Naïve	Switched	*p*	Naïve	Switched	*p*
Baseline IS/OS	12/18 (66.7)	22/24 (91.7)	0.041	6/10 (60.0)	17/20 (85.0)	0.127
Baseline ELM	9/18 (50)	9/24 (37.5)	0.418	7/10 (70.0)	11/20 (55.0)	0.429
Post-recurrence IS/OS	11/18 (61.1)	21/24 (87.5)	0.047	7/10 (70.0)	19/20 (95.0)	0.058
Post-recurrence ELM	9/18 (50.0)	12/24 (50.0)	1	7/10 (70.0)	12/20 (60.0)	0.592

*ELM* external limiting membrane, *IRC* intraretinal cysts, *IS/OS* ellipsoid junction

At baseline and after recurrence of DME, a non-statistically significant difference in terms of ELM changes was found between treatment-naïve and switched eyes in both subgroups of DME patterns (baseline: 50% vs. 37.5% in IRC and 70% vs. 55% in the mixed group; after recurrence: 50% vs. 50% in IRC and 70% vs. 60% in the mixed group) (Table 4).

### BCVA characteristics

A T test showed no differences between baseline BCVA (IRC 0.394 ± 0.287 LogMar; mixed 0.491 ± 0.334 LogMar) in the IRC and mixed groups (*p* = 0.195) and no differences between groups for post-recurrence BCVA (IRC at baseline 0.437 ± 0.296 LogMar; mixed at baseline 0.290 ± 0.191 LogMar; *p* = 0.202). In both the IRC and mixed groups, ANOVA indicated a significant time effect (*p* < 0.01) on BCVA. Post hoc tests indicated that pairwise differences in the IRC group were significant between baseline (0.394 ± 0.28 LogMar) and maximal answer (0.301 ± 0.21 LogMar) time (*p* = 0.009) and between maximal answer and post-recurrence time (0.377 ± 0.21 LogMar; *p* < 0.01). In the mixed group, a significant difference between maximal answer (0.420 ± 0.36 LogMar) and post-recurrence time (0.488 ± 0.037 LogMar; *p* = 0.015) was found.

Of note, no relevant safety issues were encountered during follow-up in this patient cohort, confirming the previously reported favourable safety profile with DEX-I.

## Discussion

Overall, the present analysis found that mixed DME eyes treated with DEX-I relapsed with only intraretinal fluid, without subretinal fluid, and at a later time compared to IRC eyes. Thus, the present data would seem to indicate that the SD-OCT characteristics that define different DME patterns at baseline may be useful in predicting the morphological features and timing of recurrence of DME. These findings further confirm the utility of SD-OCT in identifying parameters that can be predictive of a better and longer response to DEX-I.

The possibility of identifying morphological biomarkers in DME that can predict a better response to DEX-I is of substantial clinical interest. In this regard, OCT is a fast and noninvasive examination routinely used in daily clinical practice, and its application in this setting has important implications for the management of patients with diabetic maculopathy. Moreover, the present analysis suggests that patients with an IRC pattern may relapse more frequently and sooner. This provides the clinician with additional information that can help to individualize treatment with DEX-I and help in selecting patients who may be the most appropriate candidates for this therapy. For example, in patients who are predicted to relapse earlier, more frequent follow-up may be warranted in order to modulate treatment accordingly or to allow for earlier switching to another treatment, as with other therapies such as anti-VEGF [21]. One additional advantage of better prediction of relapse and time to relapse is that early implantation of DEX-I has been associated with better visual outcomes. Indeed, significantly more eyes showing a robust early response demonstrated ≥ 10-letter long-term gain in BCVA compared to eyes with poor early response [22].

Considering specific OCT features, in the present analysis, we found that mixed eyes treated with DEX-I relapsed later than IRC eyes. We could speculate that a higher percentage of mixed eyes had a recurrence of DME later than IRC eyes because the presence of SRF in mixed eyes could denote an inflammatory nature of macular oedema that could be better opposed by the action of DEX-I. These results are consistent with the study by Zur et al., which found that eyes with DME and subretinal fluid (SRF), no hyperreflective foci (HRF), and a continuous IS-OS layer responded better to DEX-I than those without these features [23]. The recurrence of DME without SRF observed in 80% of mixed eyes treated with DEX-I could also confirm the inflammatory nature of SRF, which do not reappear in the recurrence of DME after DEX-I treatment. In support of this hypothesis, previous studies have reported that higher concentrations of inflammatory cytokines in the vitreous and aqueous humour are present in eyes with SRF, thus suggesting the presence of a significant inflammatory component [4, 24]. Moreover, in our study, eyes with SRF at baseline showed greater changes in ELM than eyes without SRF (60% vs. 42.8%, respectively). This finding is of interest, considering that previous authors have reported that the integrity of the ELM seems to be a key factor in preventing fluid from passing from the outer retina into the subretinal space [25]. The post-recurrence OCT features of eyes with DME showed that eyes with SRF had more changes in both the IS/OS and ELM layers compared to eyes without SRF (86.7 and 63.3%, respectively, for mixed eyes vs. 76.2 and 50% for IRC eyes). In this regard, we speculate that SRF at baseline could be associated with damage to the IS/OS in the long term. Moreover, since there are no changes at the level of the ELM between the baseline and the post-recurrence the low rate of recurrence of SRD might depend on the timing of retreatment that was performed as soon as intraretinal fluid appeared and before the development of subretinal fluid.

DEX-I is significantly associated with improved anatomical outcomes (although not necessarily BCVA) and has been recommended as first-choice therapy for pseudophakic eyes, those resistant to anti-VEGF agents, or for patients who are reluctant to receive frequent intravitreal injections [6]. In fact, several studies have reported that DEX-I is effective for the treatment of DME, even in refractory cases that have failed to respond to other therapies, substantiating its utility in these patients [19, 26].

We also examined the difference between eyes naïve to treatment and eyes switched from previous intravitreal anti-VEGF in all cases. Interestingly, at baseline and at the post-recurrence time, treatment-naïve eyes showed fewer IS/OS changes than switched eyes in both groups, with a significant difference in IRC eyes and a statistical trend in mixed eyes. Zur et al. [23] reported that eyes with a continuous IS/OS layer respond better to DEX-I, and thus we can speculate that eyes naïve to treatment have better preservation of the IS/OS layer compared to switched eyes and thus have a better response to DEX-I. Notwithstanding, the efficacy of DEX-I in DME has been confirmed in both naïve and refractory patients [26, 27], as well as in real-world analyses [28].

Studies on prognostic indicators with the use of DEX-I in patients with DME have reported that visual and anatomical outcomes of treatment with DEX-I may be predicted by baseline visual acuity and intraretinal fluid morphology [29]. Recent data have further indicated that elevated MCP-1 aqueous humour levels and DRT pattern at baseline are biomarkers that predict future favourable anatomic response to DEX-I [30]. Thus, the present data add to the growing list of clinical markers that can help predict response to DEX-I. This is important as DEX-I is recommended in current guidelines as first- or second-line therapy in subjects with DME [31, 32] and represents a valid therapeutic alternative to other medical treatments, as also demonstrated in direct comparisons with anti-VEGF agents at 12 and 24 months [33, 34].

The present study has some limitations, such as its retrospective design and relatively low number of patients, and larger studies with additional ophthalmologic parameters are warranted to confirm our findings. Moreover, we decided not to include in the current analysis some anatomical prognostic parameters, such as hyperreflective foci and disorganization of retinal inner layers (DRIL), because of their lower application on clinical routine examination. However, it could be interesting in a further study to evaluate the association between these parameters, already described as important clinical and prognostic factors for DME [35, 36], and the pattern of DME.

We can nonetheless confirm the utility of SD-OCT in identifying parameters that can be predictive of better and longer response to DEX-I, thus reinforcing the need to further study combinations of SD-OCT and metabolic biomarkers. One such candidate for study is intracellular adhesion molecule 1, which has been shown to correlate with subretinal fluid height in DME [37].

In conclusion, most mixed DME eyes treated with DEX-I relapse without subretinal fluid and at a later time than IRC eyes. The SD-OCT characteristics of different DME patterns at baseline can help to predict the morphological features and timing of DME recurrence.
